# Is the fistula first approach still valid?

**DOI:** 10.1590/2175-8239-JBN-2022-0024-LET-en

**Published:** 2022-06-27

**Authors:** Guilherme de Castro-Santos

**Affiliations:** 1 Universidade Federal de Minas Gerais, Faculdade de Medicina, Departamento de Cirurgia, Belo Horizonte, MG, Brasil.

Dear Editor,

In the last issue of *Kdoqi (Kidney Disease Outcomes Quality Initiative),* we observed a new view of *Fistula First,* a concept much discussed in a 2006 paper^1,2^. With the evolution of materials, especially for endovascular surgery, the patency of graft access has increased. The new materials contributed greatly, especially to the procedures for the rescue of thrombosed accesses. Another important point was a more detailed knowledge of the natural history of autogenous arteriovenous fistulas, which may have a maturation rate of approximately 50% and a patency in 24 months of less than 50%^3^. Thus, it was observed that in some patients a graft access that requires a shorter maturation time is more appropriate, with the possibility of early puncture. Autogenous vein access may require a long maturation time, with the possibility of early failure and inability to puncture. People with reduced long-term survival, such as in elderly patients, would benefit from the use of arteriovenous graft. In the article: *Is the fistula first approach still valid?* ^4^, we note that in Table 2, the author was based on figure 1.5 from Kdoqi 2019^5^. Therefore, there is a difference between the contents. In the article of the Brazilian Journal of Nephrology, the author considers the indication for a graft in patients who are likely to require hemodialysis for less than one year. In the original paper, the recommendation is for patients with survival of less than one year. If we analyze the vast majority of Brazilian patients, the estimated dialysis time is more than one year. Thus, if we were to use the data from Franco^4^, we would have a inadequate indication for arteriovenous graft. Similar consideration can be made if we assume that patients with multiple comorbidities, who are older and contraindicated for transplantation, are likely to dialyze longer than young patients who do not have multiple comorbidities and are suitable for kidney transplantation. Thus, the patients who would benefit most from prostheses would be contraindicated for the procedure, according to Franco^4^. This is a very important issue that has implications for clinical practice. Brazil is a continental country with more than 100,000 hemodialysis patients and large social differences between states. Inadequate indication for arteriovenous prostheses could result in incalculable costs, both in terms of price and availability of the materials used, and increased need for procedures to maintain the functionality of these accesses.
